# Buccal region’s Kimura disease in a pediatric patient: A case report and review of the literatture

**DOI:** 10.1016/j.ijscr.2020.05.075

**Published:** 2020-06-15

**Authors:** Ahmedou Ahmed Brahim, Omar Iziki, Reda Abada, Roubal Mohamed, Mahtar Mohamed

**Affiliations:** ENT Department, Face and Neck Surgery, Hospital August, 20’1953, University Hospital Centre IBN ROCHD, street mausolée imm 10, app 9, quartiers des hopitaux, Casablanca, Morocco

**Keywords:** Kimura disease, Oral cavity, Pediatric patient, Surgical resection, Systemic steroid therapy

## Abstract

**Introduction:**

Kimura disease is a chronic inflammatory disorder that mainly manifests as a swelling in the neck region AlGhamdi et al. (2016). However, its occurrence in the oral cavity is extremely rare Lee et al. (2017). It usually affects young men of Asian race but it is rare in other races Fouda et al. (2010).

We report an extremely rare case of Kimura disease of the buccal region in a pediatric patient. The treatment consists of surgery combined with systemic steroid therapy.

**Discussion:**

The etiology of KD is unknown. Although the presence of eosinophilia and increased IgE, tumor necrosis factor (TNF)-a, interleukin (IL)-4, IL-5, IL-13 levels, and mast cells in peripheral blood, as well as in the affected tissue, were observed in patients Sun et al. (2008). No specific antigens have been identified. The optimal management strategy for KD has not yet been established. The treatment is variable Fouda et al. (2010) and Su et al. (2019). It includes surgical excision, regional, or systemic steroid therapy, and radiotherapy Sun et al. (2008).

**Conclusion:**

KD is a rare chronic inflammatory disorder of unknown etiology Fouda et al. (2010) and Kim and Szeto (1937).

The diagnosis can be only confirmed by histopathological features.

## Highlights

• Kimura disease is a chronic inflammatory disease.
• The occurrence of kimura disease in the oral cavity is extremely rare.
• The Etiology Of The Kimura Disease Is Unknown.
• The diagnosis of kimura disease is confirmed by surgical biopsy.
• Treatment of kimura disease is variable.

## 1 Introduction

This work was reported in line with the SCARE criteria and cite the following paper: Agha RA, Borrelli MR, Farwana R, Koshy K, Fowler A, Orgill DP, For the SCARE Group. The SCARE 2018 Statement: Updating Consensus Surgical CAse REport (SCARE) Guidelines, International Journal of Surgery 2018;60:132−136.

Kimura disease was described in China in 1937 with definitive histological criteria reported by Kimura et al. in 1948 [8]. Kimura disease is a rare benign chronic inflammatory disorder that typically presents with slowly enlarging, non-tender, subcutaneous swellings in the head and neck region [3]. The disease usually presents with enlarged, but painless cervical lymph nodes or subcutaneous masses in the cervical region [4]. The majority of these lesions occur in the parotid glands, submandibular glands, or neck’s lymph nodes [1,4]. There are only a few reports of Kimura disease in the oral cavity [3].The specific mechanism is unknown. However, allergic reaction, Candida infection, arthropod bite, deregulation of eosinophil dynamics and IgE synthesis, and altered systemic immune-mediated reaction have all been postulated as causative. The only way to diagnose Kimura disease is through its histopathological features confirmed by surgical biopsy. The initial treatment usually consists of medical therapy, and if it doesn’t work or if there are no signs of spontaneous resolution, then surgery would be the treatment of choice. However, radiotherapy is reserved for selected cases [4]. We report an extremely rare case of Kimura disease of the buccal region in a pediatric patient.

## 2 Case report

**Fig. 1:**
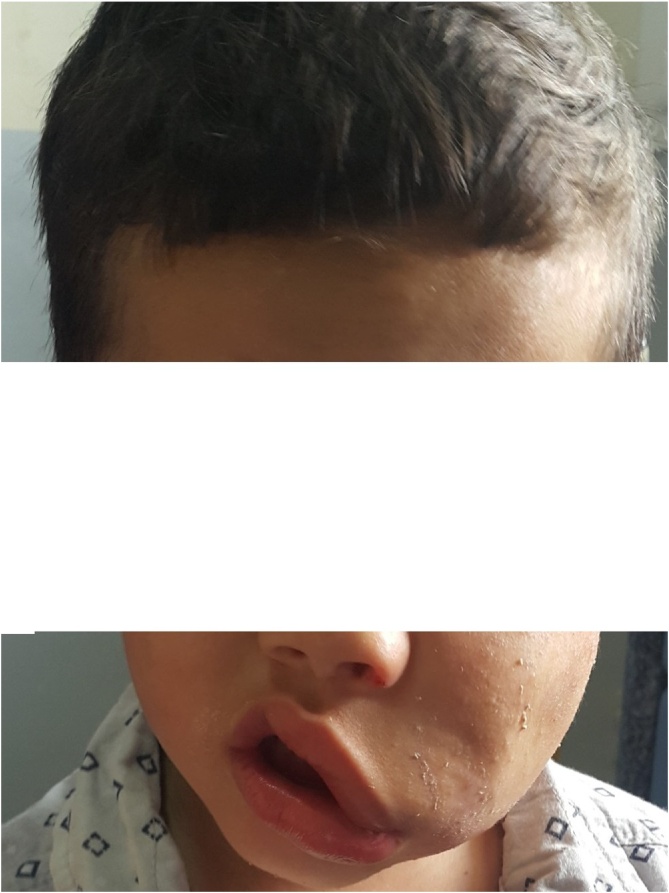
a mobile, non-tender lesion in the Left buccal region.

**Fig. 2:**
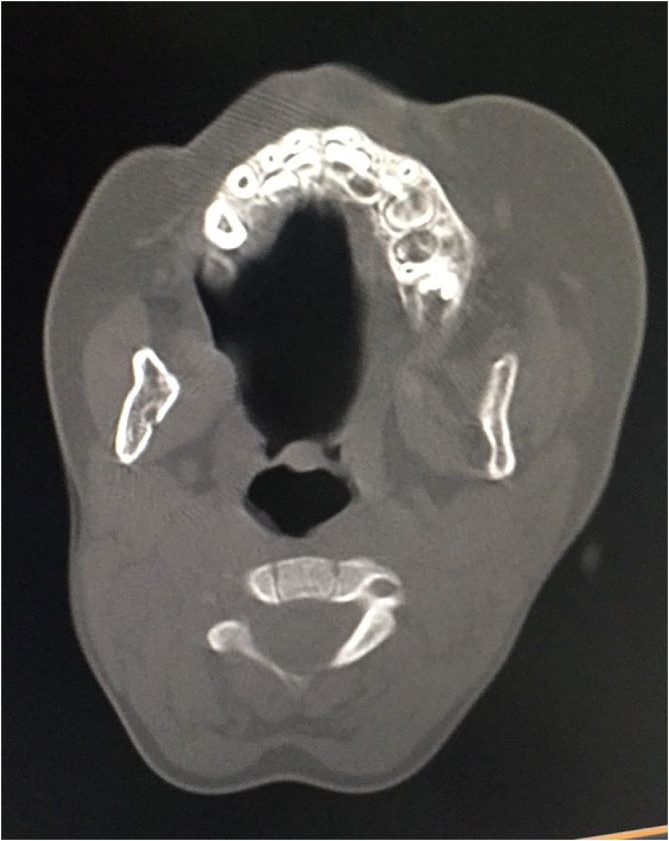
(CT) scan demonstrated a 4.5 cm nodular enhancing lesion with perilesional soft-tissue infiltration.

**Fig. 3:**
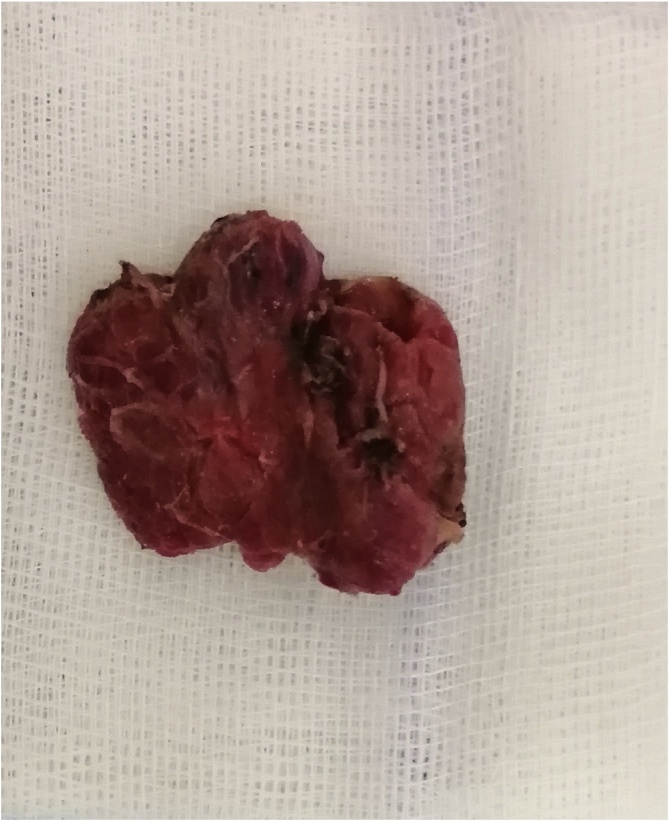
surgical specimen.

A 3-year-old patient without any particular medical history presented to our ENT department with a chronic left painless buccal swelling since birth. The clinical examination found a mobile, non-tender lesion in the Left buccal region measuring 4 cm (Fig. 1). Otoscopy, rhinoscopy as well as cervical examination did not find any abnormalities. A complete blood count showed an increased eosinophil count (50 % of total leukocyte count). Cervical computed tomography (CT) scan demonstrated a 4.5 cm nodular enhancing lesion with perilesional soft-tissue infiltration in the left buccal region (Fig. 2). The patient underwent a surgery under general anesthesia. Intraoperative exploration revealed a hard tumor attached to the surrounding structures. The tumor was excised completely without any damage to the surrounding structures **(**Fig. 3). Histopathological examination of the specimen revealed Kimura disease. Post-operatively, the patient was started on corticosteroid therapy. There has been no evidence of recurrence during the first year of follow-up.

## 3 Discussion

Kimura’s disease (KD) was first described in 1937 by Kim and Szeto [7] in the Chinese literature as “eosinophilic hyperplastic lymphogranuloma” and has been known most often as KD since its description by Kimura et al. in the Japanese literature in 1948 [8]. This rare disease is endemic mostly in many parts of Asia (Japan, China, Indonesia etc.). Although it has been reported in Europe and America [5]. KD is a rare, chronic inflammatory disorder of unknown etiology [1,7]. It usually presents in young Asian males with painless soft-tissue masses in the head and neck region [1,7]. However, the occurrence of KD in the oral cavity, similar to our case report, is extremely rare [8]. Because of its rarity in Western countries, both clinicians and radiologists are not familiar with some pathognomonic findings of this disorder, leading to unnecessary diagnostic tests and investigations [1].

The incidence of KD with coexisting renal disease ranges from 10 % to 60 %, with two-thirds of patients with nephrotic syndrome [3]. Nephrotic syndrome is the most common presentation of the systemic symptom in KD and can be associated with normal renal function. The proteinuria usually occurred months to years after the onset of the disease. A variety of renal histology disorders have been described, with membranous glomerulonephritis being the most common lesion [6]. However, the pathogenesis of both disorders is still unknown [3]. Some authors have explained that renal impairment may be probably due to immunocomplex-mediated damage, such as interleukin, cytokines, or T-helper immune response [3]. In our case, we did not detect any form of proteinuria or renal dysfunction. The etiology of KD is unknown. Although the presence of eosinophilia and increased IgE, tumor necrosis factor (TNF)-a, interleukin (IL)-4, IL-5, IL-13 levels, and mast cells in peripheral blood, as well as in the affected tissue, were observed in patients [5,9]. No specific antigens have been identified. There are some theories emphasizing the role of autoimmunity, allergy, neoplasm and parasite infestation as risk factors for KD [5]. There are so many differential diagnosis including inflammatory and neoplastic disorders, tuberculosis, cylindroma, dermatofibrosarcoma protuberans, Kaposi's Sarcoma, pyogenic granuloma, and other infections causing lymph node enlargements such as toxoplasmosis that clinicians should keep in mind [1].

On one hand, radiological assessment, such as ultrasound, CT scan, and magnetic resonance imaging, are helpful to identify the extent of the disease, and they are useful for surgical management [2]. On the other hand, their findings are variable. There are no pathognomonic radiological signs due to variable degrees of vascular proliferation and fibrosis but heightened lesions surround the parotid gland, with lymph node enlargement is a distinctive feature [4]. Hence, the diagnosis of KD is confirmed by surgical biopsy, which is characterized by angiolymphoid hyperplasia with eosinophilic infiltration [2,6].

The optimal management strategy for KD has not yet been established. The Treatment is variable [1,2]. It includes surgical excision, regional or systemic steroid therapy, and radiotherapy [3]. Surgery has been considered as the gold standard treatment for KD, but recurrence is possible [2]. Different medications have been used with a response ranging from mild improvement to a complete remission or even cure of the disease [4]. Those medications include corticosteroids, cyclosporine, cyclophosphamide, and loratadine [1,2,7].

The longest remission period of more than 6 years, was reported after the use of intravenous immunoglobulin in combination with prednisone [8]. Radiotherapy is used to treat recurrent or persistent lesions with better rates for local control, but it has a lot of side effects that limits its use as primary Modality [4]. In our case report, we performed complete surgical excision of the lesion combined to corticosteroids [2]. The disease has an excellent prognosis, although it may recur locally [1]. No malignant transformation has never been documented. However, recurrence of the disease frequently occurs in 25%–40% of patients after surgical, radiation, or steroid therapy [3].

## 4 Conclusion

KD should be considered as a differential diagnosis of head and neck swellings in the general population not only limited to Asian male adult patients as it was historically known. There are no clinical, radiological pathognomic diagnostic criteria. The diagnosis can be only confirmed by histopathological features.

## Declaration of Competing Interest

The authors declare having no conflicts of interest for this article.

## Funding

None.

## Ethical approval

I declare on my honor that the ethical approval has been exempted by my establishment.

## Consent

Written informed consent for publication of their clinical details and clinical images was obtained from the patient’s parents.

## Author contribution

Ahmed Brahim Ahmedou: Corresponding author writing the paper.

Iziki Omar: writing the paper.

Salama khadija: writing the paper.

Radhy Med Hafed: writing the paper.

Sami Rouadi: study concept.

Reda Abada: study concept.

Mohammed Roubal: correction of the paper.

Mohammed Mahtar: correction of the paper.

## Registration of research studies

researchregistry2464.

## Guarantor

DR AHMED BRAHIM AHMEDOU.

## Provenance and peer review

Not commissioned, externally peer-reviewed.

## References

[1] Fouda Mohamed Ashraf, Gheith Osama, Refaie Ayman, El-Saeed Mohamed, Bakr Adel, Wafa Ehab, Abdelraheem Mona, Sobh Mohamed (2010). Kimura disease: A. Case report and review of the literature with a new management protocol. Int. J. Nephrol..

[2] Su Sensen, Chen Xin, Li Jia, Yu Jinyu, Zhang Li (2019).

[3] Lee Dong Hoon, Kim Ga-Eon, Yang Eunmi, Yoon Tae Mi, Lee Joon Kyoo, Lim Sang Chul (2017). Kimura disease of buccal region in a pediatric patient with nephrotic syndrome A case report. Medicine.

[4] AlGhamdi Fares E., Al-Khatib Talal A., Marzouki Hani Z., AlGarni Mohammed A. (2016). Kimura disease. No age or ethnicity limit. Saudi Med. J..

[5] Sun Q.-F., Xu D.-Z., Pan S.-H., Ding J.-G., Xue Z.-Q., Miao C.-S., Cao G.-J., Jin D.-J. (2008). Kimura disease: review of the literature. Internal Med. J..

[6] Tseng Cheng-Feng, Lin Hsin-Ching, Huang Shun-Chen, Su Chih-Ying (2005). Kimura’s disease presenting as bilateral parotid masses. Eur. Arch. Otorhinolaryngol..

[7] Kim H.T., Szeto C. (1937). Eosinophilic hyperplastic lymphogranuloma. Comparison with Mikulicz’s disease. Chin. Med. J..

[8] Kimura T., Yoshimura S., Ishikawa E. (1948). On the unusual granulation combined with hyperplastic changes of lymphatic tissue. Trans. Soc. Pathol. Jpn..

[9] Shuichi Inada, Shoso Yamamoto, Hiroyuki Kitaura, Takuso Yamura (1977). A case of EOSINOPHILIC lymph folliculosis of the skin (KIMURA’S disease). J. Dermatol..

